# Barriers to and facilitators of ethical encounters at the end of life in a nursing home: an ethnographic study

**DOI:** 10.1186/s12904-022-01024-0

**Published:** 2022-07-23

**Authors:** Bodil Holmberg, Tove Godskesen

**Affiliations:** 1 Department of Health Care Sciences, Palliative Research Centre, Marie Cederschiöld University, Box 11189, 100 61 Stockholm, Sweden; 2 Centre for Research Ethics & Bioethics, Department of Public Health and Caring Sciences, Uppsala University, BMC, Box 564, SE-751 22 Uppsala, Sweden

**Keywords:** Bodily care, Caring ethics, End of life, Ethnography, Nursing home

## Abstract

**Background:**

Among a growing population of older persons, many affected by multiple diseases and complex needs, are cared for in nursing homes. Previous studies of nursing homes have highlighted the importance of personalised palliative care. Nevertheless, we know little about whether everyday care practice involving assistant nurses and frail older persons accomplishes ethical encounters, especially in assisted bodily care. Therefore, the aim of this study was to understand and conceptualize the encounter between residents and assistant nurses in bodily care-situations at the end of life in a nursing home.

**Methods:**

Focused ethnographic design was used. Residents and assistant nurses from one nursing home in an urban Swedish area participated in this study. Data were collected for 6 months and consisted of 170 h of fieldwork, including participant observation and interviews. Observations and digitally recorded interviews were analysed thematically. Five public community stakeholders contributed to the analysis by discussing preliminary results and clinical implications in a focus group.

**Results:**

Four themes, each encompassing both barriers to and facilitators of ethical encounters in assisted bodily care, were identified: Coping with the impact of workplace demands; Interacting in dialogue and communication; Experiencing involvement in the provision of assisted bodily care; and Adapting to good care and comfort.

**Conclusions:**

The findings suggest that accomplishing ethical encounters in assisted bodily care practice in a nursing home context has many barriers that are related to communication, relationships, and quality of care. Barriers included lack of resources, ineffective communication, and work values, which hinder ethical encounters. Nevertheless, moral sensitivity, genuine interest in resident engagement, and collaborative practices facilitated ethical encounters and are thus central to person-centred care. Uniquely, assistant nurses must be aware of their responsibility for performing their tasks in response to residents’ vulnerability. We therefore suggest that moral deliberation over issues of communication, compassion, decision-making, and behavior, with particular consideration for the care relationship. To further improve the quality of care, organisations must provide resources for the building of relationships, as well as time for assistant nurses to recover after long shifts. Additional research is warranted, including implementation of ethically grounded palliative care.

**Supplementary Information:**

The online version contains supplementary material available at 10.1186/s12904-022-01024-0.

## Background

The number of older persons is increasing worldwide – a development expected to continue for decades [1]. Many of the oldest have developed multiple diseases and require significant help from others. At the same time, the number of nursing homes in Sweden has decreased, following the adoption of a ‘care at home’ principle: the notion that older persons are encouraged to stay in their homes for as long as possible [2]. Thus, access to nursing homes is only granted to the oldest and most fragile. Calculations from the Swedish palliative care register show that just over half of older persons die within 6 months of moving into nursing homes [3]. These older persons generally need help with assisted bodily care (ABC) several times during the day and night, assistance that is mostly provided by assistant nurses (ANs). ABC includes assistance with dressing and undressing, mealtimes, transfer and personal hygiene. It also comprises aid with things considered to be important, but which are difficult to fulfil when affected by inhibited bodily capacity. Further, as ABC concerns the body as a whole, it also aims to stimulate the mind [4].

Older persons, the very recipients of ABC, typically describe its meaning as being twofold [4]: it either signifies pleasure and well-being, or imprisonment and dejection. The difference emanates from whether knowledgeable and gentle hands provide the ABC in an atmosphere of friendship and humour that pushes the care needs into the background, or if it is provided in a hurry, with recklessness, associated with long waiting times and without the recognition of personal preferences. The ideal relationship with ANs has been described as one that is close and friendly, characterised by calm and respectful behaviour, where ANs learn about the older persons’ preferences and adapt their ABC accordingly [5]. In any case, the role of being a recipient of ABC is not optional.

Nursing homes are highly dependent on ANs to provide ABC to residents. ANs are the largest group and account for 6 out of 10 employees, and thus outnumber any other occupation employed in nursing homes in Sweden [6]. However, the organisation of nursing homes is suffering from low staffing levels [5], with few nurses and physicians. Consequently, nursing homes rely on a workforce that usually has low education levels [7]. Further, ABC work has generally held a low social status and is viewed as ‘dirty work’ in its dealings with bodily fluids and unpleasant odours [8]. In addition, it originates from the tradition of being low-paid women’s work.

In health care, the four principles of biomedical ethics, as outlined by Beauchamp and Childress [9], have become the cornerstone of biomedical ethics. These include: respect for autonomy, beneficence, non-maleficence, and justice, and are built on both deontological and consequentialist ethics. However, other bioethical theories are also relevant and important to healthcare practices, for example, virtue ethics, which focus on professionals’ moral character, rather than their actions, in order to bring about good consequences. In addition, care ethics place special emphasis on mutal interdepence, reciprocal relationships, sensitivity, vulnerability and dependence, which is applicable to analyses of ethical issues and moral dilemmas in nursing homes [10]. The concept of care ethics (or the ethics of care) was initially developed by psychologist Carol Gilligan [11]. Care ethics add to consequentialist and deontological theories and their premise that moral decision-making should be rational and logical and focused on universal and objective principles or rules. Gillian and the philosophers Virgina Held and Ned Noddings instead emphasised a feminist normative theory focused on a “voice of care” [12, 13]. From this perspective, relationships are seen as being essential and, by contextualising normative decisions, it stresses the well-being of care givers and care receivers situated in a network of social relations. Human beings are considered to be co-dependent and, for this reason, respect, protection, and care are fundamental notions for expressing such inherently caring relationships [12]. In this study, the instances described below define the content of an ethical encounter. Such an encounter requires ABC that pays attention to and adapts to older persons’ physical needs, preferences in ABC and their life experiences. Thus, it is of utmost importance for ABC improvement that ANs get to know the older persons’ life stories and establish trustful relationships. According to Held [12], trust is a fundamental value in caring and care activities, and a prerequisite for an ethical encounter. This complies with the mandate that palliative care needs to be ethically grounded, thus building strong and meaningful relations [14]. Consequently, every moral choice or ethical issue involving others is perceived as being situated in a network of relational interactions nurtured by communication [12]. This can be labelled as relational care, where ANs experience themselves as having a responsibility and willingness to encounter older persons in situations of extreme vulnerability, intending to maintain their well-being and promote their dignity. Such a focus on relationships furthers empathetic involvement and implies a particular responsibility towards those who are powerless rather than powerful. In clinical encounters, compassion, sensitiveness, vulnerability, autonomy, integrity and respect are important notions [15], not least at the end of life. Ethical sensitivity, defined as a caring response or intuition about others’ feelings or being moved by, or identifying with others’ distress, is central to caring encounters, thus essential in a nursing home context [9]. In this study, palliative care refers to the point of diagnosis until death, while end-of-life care refers to the care provided in the final months of life [16].

Previous studies of nursing homes have highlighted the importance of providing personalised palliative care. Nevertheless, we know little about whether everyday care practice involving assistant nurses and frail older persons realises ethical encounters, especially in ABC. The aim of the study was to understand and conceptualize the encounter between residents and assistant nurses in bodily care-situations at the end of life in a nursing home.

## Methods

### Design

A focused ethnographic (FE) design was used. FE (also called micro-ethnography) has its roots in anthropology [17] and enables the exploration of a particular issue in a specific setting. It focuses on subcultural groups that share particular traits instead of looking at whole societies [18]. FE is a widely endorsed method in healthcare research [17, 18], and is known to be particularly suitable for research involving older people [19] and palliative care [20], as the method includes short field visits and is observational rather than participatory. The research question that guided the analysis was as follows: What are the barriers and facilitators of ethical ABC encounters at the end of life in a nursing home? Specific elements of interest were aspects of communication, relationships and quality of care.

### Setting

The participants were recruited from a nursing home in an urban Swedish area where ANs provided ABC. Only one nursing home was chosen, as ethnographic research typically requires long periods of time in the field to reach a certain level of intimacy with the participants [17]. Further, this specific nursing home housed participants diverse in their genders, ages and ethnicities. The nursing home housed 32 residents in four accommodation units comprising eight residents each. The residents lived in one-room flats, each with an adjoining bathroom and kitchenette. Each unit shared a common living room and kitchen. The residents’ median length of residency in the nursing home was 17 months (range: 14 days – 6.75 years, calculated from room-renting data from 2010 to 2017). When staffing was low, an ambulatory assistant nurse aided temporarily in all units, following a pre-planned schedule (Table 1), with weekend afternoons excluded.

**Table 1:** The distribution of ANs during a 24-hour period

Shift specifics	Number of ANs/ shift
Daytime weekdays from 7 am until 4 pm	2
Evening weekdays and weekends from 4 pm until 9 pm	1 + 1 ambulatory, covering all units
Weekends from 7 am until 12 noon	2
Weekends from 12 noon until 4 pm	1
Nights from 9 pm until 7 am	2 covering all units

The nursing home had one registered nurse working during the day; otherwise, one was available on-call. The nurse did not participate in the provision of ABC, but supervised staff and acted as a consultant. One General Practitioner was present for three hours once a week, otherwise available on-call. The nursing home practised standard times for the provision of ABC. The standard time for a shower was 30 min. The standard times for feeding/motivation (to eat) was 25 min, and the standard times for morning/evening help was 20 min and included dressing/undressing and toothbrushing. A visit to the toilet had a standard time of 15 min.

The daily lives of the residents in the nursing home followed routines for meals, medication, personal hygiene, transfer and periods of rest. Volunteer participation in regular activities such as singing, bingo and gymnastics was offered.

### Recruitment and participants

Written information about the study was first sent to the nurse-in-charge, who provided verbal and written information about the study to the residents who met the inclusion criteria: permanently living in the nursing home, affected by multimorbidity (> 2 chronic diseases), able to understand verbal and written information, able to express themselves and interact in a conversation, and in daily need of ABC. Next, the researcher visited the residents, offering further verbal information about the study and its purpose. If needed, the residents were offered time to consider participation. Of the twenty-two eligible residents, eighteen agreed to participate. Thereafter, two residents affected by severe cognitive deterioration were excluded. This study comprised observations with follow-up interview/s. As some residents could not always cope with being interviewed after an observation, four were subsequently excluded and twelve residents were ultimately participated in the study (Table 2).

**Table 2:** Participant characteristics

	**Residents (*n*=12)**	**Assistant nurses (*n*Residents (=16)**
**Sex**		
Female	10	16
Male	2	-
**Place of birth**		
Sweden	11	13
Europe	1	2
Overseas	-	1
**Age**		
20-30	-	2
31-40	-	3
41-50	-	3
51-60	-	6
61-70	-	2
71-80	-	-
81-90	5	-
91-100	7	-
**Mean Age**	90.1	47.4
**Mean years of experience as assistant nurse**		15.8
**Degree of residents’ bodily help needs**		
Totally dependent	1	
Help with shower	11	
Manges to shower with support	1	
Manages to use the toilet	3	
Help using the toilet	9	
Manages to brush teeth/ face, shave	4	
Help to brush teeth/ face, shave	8	
Manages to dress/ undress	0	
Help to dress/ undress	12	
Manages to eat unsupported	4	
Help with food	8	
Help with medicines	12	
Manages to walk with roller	4	
Help to move in wheelchair	6	
Manages to move independently in wheelchair	2	
**Participation in observations**	12	15
**Participation in interviews**	7	13

 Next, the researcher attended workplace meetings where ANs were provided verbal and written information about the study and its purpose. Of the thirty potential participants who met the inclusion criteria of having at least 1 year’s experience of working in a nursing home, twenty-eight ANs agreed to participate. Because of time constraints, some ANs did not have time to be interviewed after an observation. Finally, sixteen ANs participated in the study (Table 2).

### Data collection

The first author (BH) conducted 170 h of fieldwork observations over 6 months in 2017 by observing ABC situations during both the day- and night-time and performed interviews with participants. During the fieldwork, the researcher sometimes interacted socially, but not in the ABC activities. As the focus of this study was encounters between residents and ANs in ABC situations, we followed Hackett, who argues that encounters and interactions should be the focus of ethnography, rather than participants’ individual actions [18]. As an observer, the researcher also partly participated in these encounters, following assistant nurses and residents as they interacted [17, 18]. The participants were followed from 3 to 8 h a day, with the aim of understanding the scope and nature of their interrelationships in ABC situations, as they often express their experiences both verbally and emotionally, as reflected in their body language [20]. A two-part protocol was used, where the first part described demographic data such as location, time and date, and the latter described the interactions in free text. While observing, the researcher took notes and transcribed them immediately afterwards. The ANs’ demanding schedule, and residents’ condition, mood and activities, guided the most suitable choice of time and place for holding follow-up interviews (*n* = 27). In this study, observing multiple ABC-situations within a variety of perspectives was essential, as it provided a unique opportunity to reveal insights into the perspectives of residents and ANs. Therefore, we used observations, combined with follow-up interviews, which allowed us to elicit further understanding of what we observed. The data comprised sixteen observations, of which nine included follow-up-interviews with both a resident and assistant nurses. One observation included a follow-up interview with a resident, and six included follow-up interviews with an AN. The mean time for follow-up interviews was about ten minutes. All interviews were audiotaped and transcribed verbatim.

Triangulation with stakeholders is a method used for trustworthiness and to enhance the validity and rigour of the data analysis in qualitative research [21] and ethnography [22]. Stakeholder engagement can increase transparency, guide the research process, and result in recommendations for clinical practice [23]. In this study, a number of stakeholders were invited to participate, as they offered valuable insights on important aspects by eliciting further understanding of the initial observations. The stakeholders participated in a focus group discussion that took place online using a digital meeting software platform: an 80-year-old with ABC experience, an assistant nurse working in a nursing home, a nursing home manager, a registered nurse with medical responsibility in municipal care, and an elected member of the municipal council. All stakeholders consented to participate in a focus group discussion. Before the discussion took place, they were instructed to read the preliminary findings and identify those they deemed to be most important, whether these seemed trustworthy from their perspective, and to pinpoint clinical implications. The focus group discussion was audiotaped and lasted for 90 min.

### Data analysis

Consistent with the FE approach, data analysis started simultaneously with data collection [17]. The data were analysed using inductive thematic analysis [24]. The first step of analysis involved reading and re-reading interviews and field-note transcripts to become immersed and intimately familiar with the content. Second, the entire dataset was again scrutinised to complete an initial coding, identifying important and relevant features of the data. Third, all codes were scrutinised, and significant broader outlines of meaning (themes) were identified. This was the most extensive process, going back and forth between the raw data, codes and the initial mind-map of themes from the reading phase (see S1). This data comprised 181 pages (A4) of single-spaced text. Fourth, themes were rechecked against the dataset to ensure that the data told a convincing story and that the themes answered the aim. To ensure this, all the collated data within each theme were summarised in new documents, further discussed, refined and reviewed several times. Fifth, a more comprehensive analysis of each theme was attempted, aiming for a focus and a ‘story’ relating to each theme. An informative title for each theme was determined. The sixth and final phase included writing the paper.

### Ethical considerations

Respect for the participants’ integrity was crucial during the fieldwork. To avoid confusion about the observers’ presence during ABC, the residents’ cognition was observed and evaluated continuously. Further, the researcher wore a badge, printed with her name and the word “researcher”, in order to confirm her role and to prevent confusion. If residents were unsure about the researchers’ presence, or if they had misunderstood or were affected by sudden memory loss, the study information was repeated, and renewed oral consent was confirmed before observing. The collected data were handled according to the EU General Data Protection Regulation (2016/679). During the observations and field notes, characteristics were immediately changed, such as names, dates, locations, and relationships. All identifiers were removed from the transcripts and data were stored securely in a password-protected computer.

## Results

In the analysis, four themes elucidating barriers to and facilitators of an ethical encounter in ABC emerged: (1) Coping with the impact of workplace demands; (2) Interacting by dialogue and communication; (3) Experiencing involvement in the provision of ABC; and (4) Adapting to good care and comfort (Table 3).

**Table 3:** Themes with examples of barriers and facilitatiors of ethical encounter

Themes	Barriers	Facilitators
Coping with the impact of workplace demands	- Lack of resources - Time restraints	- Mutual collaborations - Redistribution of staff
Interacting by dialogue and communication	- Making derogatory comments about residents - Harsh and dismissive language	- Warm communication tone with humourous elements - Comforting communication
Experiencing involvement in the provision of ABC	- Interupting residents’ independent acts - Ignoring residents’ clothes and meal preferences	- Caring activities in line with residents’ preferences - Allowing residents to perform what is possible
Adapting to good care and comfort	- Not knowing the residents’ abilities - Not asking for residents’ preferences	- Supporting residents’ independence - Attentivness to residents’ physical needs and interests

Lack of resources, organisational factors and the dilemma of not having enough time were frequently mentioned as being obstacles to ethical encounters. This affected residents’ sense of personal autonomy and became a barrier to their involvement in decision-making and in receiving person-centered care.

The organisation of the nursing home presented several demands that challenged ethical encounters. ANs claimed they had so many practical tasks to fulfil in a short time that it was difficult to give residents the necessary attention. ANs were constantly hurrying, perceiving this to be a considerable concern, as someone was always waiting. This prevented them from joining residents’ conversations and thwarted their ambitions to make residents feel seen. It hindered their aspirations to take residents out for much-wanted walks, as ANs needed to follow strict routines. ANs were often interrupted by alarms and telephone calls, which made them forget details in ABC. Further, they lacked time to read the nursing notes to learn about changes in residents’ condition. In all, they found this created an irritable atmosphere among residents and a personal stress of conscience. They feared unexpected events, i.e., falls, which forced them to re-schedule the routines, resulting in increased annoyance among residents, who were prevented from obtaining expected and demanded help. To facilitate communication with colleagues and to hear cries from other residents, ANs claimed it was necessary to keep the doors open while helping residents with personal hygiene. Hurrying entailed them standing next to residents who were using the toilet, transferring them through the room while positioned in lifting aid equipment, and applying double incontinence pads to save time when changing them. They preferred residents to sit on the toilet while showering, as the passing of stools during the shower was time-saving. When asked about the convenience of this, an AN answered:

*Interviewer: Why does she sit on the toilet to shower? AN: It is easier if she sits on the toilet while we shower her. Interviewer: Isn’t it unusual to sit on the toilet and shower?AN: I don’t know (laughs) I can’t answer that. They are old routines or something, maybe the occupational therapist has said it? I don’t understand what is right or wrong.* (Obervation 31)

In addition to insufficient time resources, ANs mentioned that staffing levels had been cut, that the nursing home manager did not understand the relational aspects of their work, nor had they listened to their views concerning the need to expand staffing. The manager also expected ANs to be flexible and work as temporary replacement for other units. ANs critisized this, as they did not know these residents well enough to provide safe, knowledgeable and personalised care. In summary, organisational obstacles drained ANs’ joy in their work, prolonged their recovery after hard working days, and urged some to seek other types of work. One AN, working an evening shift, expressed her feelings in this way:

*Now when it is such nice weather in the evenings … instead of taking someone out for a walk to get some fresh air, we don’t have time. We just run around. I go to work happy, but come home with a bad conscience, I can’t give these people what they deserve. You can’t touch and hug or brush teeth as you should and if something unexpected happens, such as someone vomits, the other seven residents will also suffer. I am stressed almost to death. At some point we will all end up in these places, but they don’t want to improve it. I don’t want to live like this, I really don’t!* (Observation 18)

Residents pitied ANs, who were always perceived to be in a hurry. They, too, found that time pressures and low staffing levels deteriorate ABC and negatively affect the atmosphere. They claimed that the routine of showering once a week is insufficient, but hesitated to disturb busy ANs with requests for additional help. However, the situation made them fear eventually needing an increased level of ABC.

Conversely, ANs suffered from lack of time and low staffing levels, as this prevented them from being attentive, meticulous and empathetic in ABC encounters. They tried to solve these problems by planning their time, i.e., preparing sandwiches for the evening in the afternoon, to release time for unexpected needs during the evening. This planning was constantly ongoing within mutual collaboration, where ANs agreed on how to work and helped each other when needed. Further, ANs independently redistributed staff resources when residents were about to die to secure extra attentiveness from ANs who were most familiar with them. When no residents had special needs, their ambition was to consciously distribute resources equally among all residents.

Residents observed ANs’ situation with understanding, referring to their heavy workload. They expressed awareness of all residents’ similar needs of ABC, particularly during mornings and evenings. When possible, residents with fewer needs of ABC helped other residents, and did what they could to provide for themselves, i.e., making their beds. They did this not only to be active, but also to facilitate the ANs’ situation.

### Interacting by dialogue and communication

The daily dialogue between residents and ANs during ABC could pose barriers to ethical encounters, for instance, when ANs spoke to each other without including residents. These conversations concerned their ongoing working tasks or the planning of work with other residents. They also included derogatory comments about the residents’ bodily constitution, ridiculing or scoffing at them, laughing at their expense, and questioning their habits. They sometimes made derogatory comments about the residents’ significant others. When ANs were observed alone with residents, communication was hampered when residents spoke without receiving an adequate response, here illustrated in their communication after a shower:

*AN: Picks up the large towel and dries hair, face, and armpits. She asks: Do you feel dry now? The resident answers: What? and continues the story about rapes in 1940s (name of war-torn city). She says: I got through it. AN answers: Hhmm. The resident closes their eyes. AN squats and pulls on knee high socks. The resident continues their her story: I can honestly say that I pulled through it, I was never raped, but most were. They never told me, but I knew it happened, even though I was only a child. AN takes off her apron, opens the door and stuffs it into the waste basket just outside, saying: I’ll get a jumper.* (Observation 20)

ABC was rarely preceded by questions regarding residents’ perceived needs, nor questions about their preferences. If questions preceded actions, residents were not always given time to answer before the action was performed. ABC was defined by ANs commanding residents what to do, when to bend forward or lift an arm. Communication regarding the next step in the ABC-process was lacking, as was information about how long residents could expect to wait for help. ANs lied to residents by not fulfilling promises, i.e., when an AN promised a resident they would wash her genitals in the shower but immediately informed her colleague that they would not, as this had already been done in bed before the shower. Further, ANs and residents argued about drugs, i.e., concerning whether or not it was time to take medication for bowel function, or concerning the choice of clothes, where ANs neglected residents’ expressed preferences.

ANs’ communication with residents was sometimes harsh and dismissive, and they appeared to be annoyed. An example of this was when a resident asked to stay up late to watch a programme on TV but was told that this could just as well be done while lying in bed. In interviews, residents commented that reprimands and negative attitudes from ANs were humiliating, while ANs claimed to be aware that their negative attitudes in communication caused anxiety for the residents.

Conversely, a polite and mutual relationship between residents and ANs facilitated an ethical encounter in ABC. For example, when ANs posed questions about residents’ condition and asked whether they wanted help, or when asking for residents’ preferences in ABC before acting, followed by clear instructions on how they could collaborate. Here, ANs carefully informed residents of planned actions, and included opportunities for shared decision-making.

In interviews, ANs emphasised the need for having relaxed conversations with residents to strengthen their mutual relationship. They described intimate moments characterised by trust and confidentiality as a privilege in their work. Mutual relationships were observed when residents and ANs performed confidential conversations on topics chosen by the residents. This was strengthened when a warm and lighthearted conversational tone with humourous elements created a dialogue where the residents were listened to. Smiles and eye-contact occurred frequently, as did ANs’ encouragement when residents performed actions they perceived to be difficult.

ANs were seen to comfort residents when they expressed sadness. They equalised their relationship with residents by paying attention to new haircuts, or joking about their own bodily signs of ageing. ANs communicated empathy for the residents, and demonstrated an interest in their feelings by asking whether performed actions were satisfactory. In mealtime situations, ANs facilitated residents’ communication by including silent residents. This was claimed to be important for making residents happy. An AN expressed her devotion for communication with residents in this way:

*This is the world’s best job!! I say this to all our students. In what other place can you laugh so much, sing, dance, tell cheeky stories? You’ve got the lot…all these positives you get with this job.* (Observation 9)

Within this facilitating dialogue, residents performed as active and equal partners, who announced their preferences in ABC, told ANs when to stop an activity and clearly announced discontentment with the performance of ABC. They encouraged and praised ANs’ work.

### Experiencing involvement in the provision of ABC

Situations preventing residents from involvement in ABC were observed. This hindered ethical encounters, for instance, when ANs knocked on residents’ doors without awaiting an answer before entering. Likewise, when residents aimed to act but were preceded by quicker ANs, or when residents refused food or medications but were fed anyway, not knowing that the drugs were hidden within the food. This also occurred when residents had decided what clothes to wear but were dressed in other garments chosen by ANs. Prevention of involvement could entail physical inconvenience to residents, here exemplified by a resident getting helped to get dressed:

*AN: I couldn’t find any cotton knickers/…/ she squats, puts on a pair of incontinence pants, pulls them up. /… / She supports the resident who gets up. AN pulls up the pants. Resident: I can’t wear these, they are too tight. AN: I can cut them open a bit, there aren’t any others. She pulls up the skirt and fastens it. Resident: It’s a bit tight. She tugs at the pants, saying: These aren’t right, they’re way too small. AN: Yes. The resident goes out from the toilet, towards the armchair. She says: I can’t sit down, that’s how tight they are. She sits down. AN brushes her hair /… / Resident: You must ask if they have any underwear for me. AN answers: Yes. AN goes in the bathroom and comes back with the upper set of false teeth. A: Here are your teeth.* (Observation 20)

Residents expressed their lack of influence on decisions about meals, as their desired food was not always available. Shower routines entailed that residents who declined a planned shower had to accept that it would be postponed until next week. Likewise, residents being prepared to shower had to accept it was cancelled altogether when unexpected events forced ANs to re-schedule their tasks.

The residents’ approach to this was cautious. They accepted their inhibited involvement, did not remind ANs when they forgot certain personal routines, such as applying deodorant. This cautiousness is exemplified by a resident who experienced tenderness in her genitals, wishing ANs to arrange her trousers loosely:

*Resident: If there is something that is sitting against (the groin), it hurts. You have to take your trousers and try to shape them so they don’t hurt you, but they are all different too, the staff. Some are careful and gentle, but some are not… Interviewer: Do you say anything? Resident: No /… / some won’t believe it hurts* (Observation 7).

Residents also refrained from being involved by transferring decisions to ANs, such as a resident who wanted her stoma bag emptied but instead accepted keeping it full, as the AN disagreed with the need. Residents claimed that their approach depended on their unwillingness to be a burden to ANs.

Again, situations characterised by resident involvement may be described as those that facilitate an ethical encounter in ABC. This was observed when residents decided how to perform ABC-situations, according to who does what, and what soaps and lotions to use. ANs claimed to respect residents’ desires to accomplish certain moments independently, although difficult and time consuming, as resident involvement was considered important and beneficial. Thus, they strived to preserve their abilities and skills for as long as possible. To further facilitate residents’ involvement, ANs asked residents to judge the water temperature when showering, and were willing to help when residents wanted to make appointments with the hairdresser or podiatrist.

Residents’ involvement comprised performing activities in ABC, doing all that they still could, such as holding a paralysed arm during transmissions. They claimed to enjoy making decisions for themselves, concerning what to wear and when to attend social events. Involvement provided them with a feeling of luxury, here described by a resident who had chosen to join a tour in a carriage behind a bicycle:

*Resident: I can tell you, it was lovely! We were by the pool where we used to bathe, many times. It was lovely! Interviewer: How did it feel to sit there? Resident: It was nice… You just can’t understand it is real. It just feels good to be out in nature… when you used to cycle so much before.* (Observation 12)

An ethical encounter in ABC could also be facilitated when ANs inhibited residents’ involvement. For instance, residents who found it difficult to choose what to wear gratefully transferred this task to ANs. Another way of inhibiting residents’ involvement positively was when ANs found time to aid residents with minor help needs, to make them feel cared for and a bit spoiled.

### Adapting to good care and comfort

ANs’ lack of adaption to good care challenged an ethical encounter in ABC. Even long after residents’ admission to the nursing home, ANs lacked knowledge of residents’ preferences, finding it difficult to interpret their behaviour. This was exemplified by an AN, commenting on a resident:

*AN: I think I had worked here for one or two weeks before I realised that [Resident] could actually talk, because she didn’t say a word. I like thought she just couldn’t speak.* (Observation 16)

ANs’ attitudes could also entail the perception that residents lacked an opinion of their own, i.e., concerning what to wear, thus it was unnecessary to ask them about such things. Alternatively, it urged residents to continuously repeat information about their preferences. ANs’ ignorance was observed when residents’ health status changed more rapidly. For instance, in an observation of a dying resident who was semi-conscious, incessantly groaning and calling out from her bed from the early morning, the ANs assumed this resident was about to die soon but did not prioritise contacting a nurse until their workload was expected to lighten soon after lunch. Nor did they search for knowledge about the resident in the nursing notes. Instead, they woke her up to wash her, administrate medicines orally, and feed her, which resulted in the resident vomiting. Afterwards, they left her alone, expecting her to press the alarm button if needed. Other examples of lacking adaption were when ANs washed residents with cold washcloths or initiated ABC-activities on residents who were still asleep. Further, when they omitted to wipe the bottom of residents who had micturated while showering. Sometimes, ANs were heavy-handed and did not adapt their behaviour until residents groaned or gasped. Lack of attentive adaption also negatively affected residents who had limited need of ABC, here exemplified by a resident:

*Resident: They completely forgot me. Because I am not that unwell, I am actually here because I’m just old and they forgot to come and say anything. I didn’t know what to do, I was upset that they never came… Yeah, you feel a bit worthless when they never come.* (Observation 32)

Residents appeared to facilitate ANs’ lack of adaption by not opposing them in their ABC-activities. If the shower water was too hot, they claimed that they reminded themselves that their main goal is to become clean. Moreover, residents described a voluntary adaption to the routines, i.e., by going to bed early, while plenty of help was available in the ward, and by accepting prolonged waiting times when ANs were busy helping others.

Conversely, an ethical encounter in ABC situations was facilitated when ANs knew how to provide good care, were aware of residents’ preferences and abilities, and compliantly adapted their provision of ABC, for instance, when residents performed certain moments independently, such as eating or brushing teeth, and ANs calmly awaited the moment of further needed help, or when they efficiently passed soaped washcloths to residents who independently washed easily reached body parts in the shower. Adaption also included ANs altering their actions during ongoing ABC, i.e., by fetching alternative clothes that the residents asked for. Thus, they used their knowledge to adaptively collaborate with residents.

ANs’ adaption comprised attentiveness to variations in residents’ daily status, such as preventing accidents by reaching out a hand to residents who wobbled, or steadying residents’ feet during transmission. ANs underlined a need to attentively and efficiently support residents’ wishes by interpreting their varying moods. Thus, they noticed signs of stiffness, chills, tiredness or loneliness and unsolicitedly adjusted residents’ positions in bed or in wheelchairs, fetched blankets, postponed breakfast, or provided extra touch and company. This was also shown by personalised preparations, i.e., putting pieces of tissue in strategic places for a resident who was affected by a dripping nose. Again, ANs claimed such tasks were important to respect and perform, as they assumed residents missed their ability to manage independently. Their adaption also included overruling their norms, i.e., performing showers very quickly with residents who preferred it that way, irrespective of their own ambitions to perform them more slowly. Also, they avoided changing scheduled shower times for residents with cognitive failure, as they might not comprehend the reason. Moreover, they were seen to adapt to residents’ unique interests, i.e., when helping them complete forms concerning political elections, or by being compliant with residents’ preferences of appearance, i.e., folding up long sleeves or applying nail polish. The adaptiveness also comprised guarding integrity, by closing doors when residents were naked, or looking away while shy residents undressed.

Residents described ANs’ adaptiveness as an attentive kindness and cheerfulness that made them feel as though they were being taken seriously. This loving encounter could compensate for losses experienced earlier in life, here described by a resident:

*Resident: You get love from the girls here, you know. They come and cuddle you and… Mother wasn’t very generous with that. We never got to sit on our mother’s lap and cuddle, she had too much to do. Interviewer: But you get to now? Resident: Yes, now I do! Now I do with the girls here.* (Observation 16)

Residents were also adaptive by accepting that staff would see them naked in vulnerable situations, and by carefully attending and following ANs’ instructions in ABC-situations.

## Discussion

The aim of this study was to explore barriers to and facilitators of ethical encounters in assisted bodily care at the end of life in a nursing home. Based on the significant results, this discussion elucidates three important areas concerning ethical encounters in ABC.

### Being under the influence of workplace practices and values

Workplace practices and values often hindered ethical encounters. ANs mentioned that time pressure and low staffing levels forced them to strictly follow routines, which hindered them from providing good, personalised ABC. This aligns with earlier research, describing a lack of sufficient resources regarding time and staffing in nursing homes [9, 25], which urges ANs to focus on tasks, irrespective of the holistic needs that they perceive and to which they wish to attend [26]. The results showed that ANs provided care they would never want for themselves. Other research describes ANs as marginalised, undervalued and left out of decision-making [27]. Time pressures make them feel torn between their duties to residents’ needs, which inhibits their time to build relationships, and sitting and talking to residents is not considered work by colleagues. The common understanding in nursing homes is that a clean unit with clean older persons signals competent assistant nurses [28]. On the other hand, the results showed that the opposite would jeopardise the fragile system of routines on which the system depends. Small disturbances in the schedule causes negative consequences for all residents in the unit, especially when residents deteriorate rapidly. This is in line with other research that highlights ANs’ fear of neglecting serious symptoms among residents when leaving to help another [29]. This adds ethical challenges to the practice. One conclusion is that particular attention should be paid to the workplace culture and values, or, in Jeanette Pols’ terms, the promotion of contextual reflexivity as a practice, with a focus on good care and awareness of ethical aspects in everyday work [28]. Alternatively, this study shows that ANs independently managed to redistribute staff resources when needed and prioritised sitting down and socialising when possible. This indicates the presence of an ethical sensitiveness in a complex working environment, one that is not conducive to relationship-building.

### Importance of mutual relationships

Although the observed care provided was primarily ABC, it often yielded highly multifaceted ethical situations. The results showed that an ethical encounter was possible, regardless of barriers caused by time pressures and low staffing levels, that is, when ANs were focused on residents’ preferences and acted accordingly. This requires ANs to have a moral sensitivity, being truly interested in residents’ lives and preferences and in reducing their problems, fulfilling their needs, and realising their dreams [12], thus, striving to establish relationships that are open to residents’ desires and needs in an unselfish way. Here, care ethics could serve as a suitable point of reference, where the motivation is caring for persons who are dependent and vulnerable, and where compassion, sensitiveness, trust and respect are important notions [10–12]. However, the results also showed that residents also need to be engaged, by declaring their preferences and guiding the ANs on how they want ABC to be performed. Thus, an ethical encounter in ABC requires a mutual relationship. In this study, residents’ mutuality appears from their concerns about assistant nurses’ heavy workload, and their expressed appreciation of their work. If this relationship is indeed supposed to be mutual, the residents are therefore responsible for being honest and willing to reveal themselves to the ANs. This is the residents’ major contribution to the dual relationship needed for an ethical encounter [12]. Accordingly, the ANs’ actions to equalise the relationship by joking on their own behalf may be described as attempts to facilitate the residents in feeling relaxed. Furthermore, mutual cooperation, and residents’ participation in ABC, facilitates the relationship.

However, sympathy alone is not enough. The philosopher Martin Buber [30] argues that, in an ethical encounter, both participants need to be interested in reciprocal relationships, receiving the other person as a subject, and a crucial partner in one of the scenes of their own life [30]. Buber refers to this as an I-Thou relation, where humans meet each other’s essence, extending from the standpoint of human love. This sort of ethical encounter may be important to older persons in nursing homes, in this study exemplified by the older woman who found that the ANs’ love replaced her mother’s love, which she had missed her whole life. The opposite of an I-Thou relation would be an I-It relation, where the other person’s subjectivity is overlooked and the person is objectified [30]. In such cases, there are no options for an ethical encounter. I-It relations occurred in the results, i.e., when a resident spoke about her war memories without receiving an adequate response. Apparently, on that occasion, the AN was not fully present, nor interested in the person she was helping. Her only interest was the practical task that she was there to fulfil; thus, the resident was being objectified. Consequently, according to Buber, there was no relationship in that encounter, which hindered an ethical encounter, at least in terms of paying attention, and adapting to the older person’s physical needs and preferences [30].

ANs in this study were observed speaking about residents in negative terms. Sometimes they spoke harsh and unkind words openly. Such negative attitudes towards residents have been subject to several studies previously [27], and “ageism” has been identified as a factor [31]. When communication is task-focused and stressful, ANs might not realise that negative attitudes may lead to inequality, marginalisation and discrimination [31, 32]. This raises ethical issues that need to be addressed among ANs in nursing homes. Moral sensitivity, i.e., awareness of how one’s actions can affect others, needs to be promoted. Supervision and educational interventions could be instruments for this, emphasising the need to consider the older person’s values and uphold their autonomy [33]. Once ANs have been made aware of their responsibilities and understand how their ways of communicating risk letting residents down, the moral demand to act in a justifiable way has been established [9, 14]. Thereby, they become responsible and accountable for whether they perform their tasks in response to residents’ vulnerability.

### The necessity of trust

When ANs are compliant with residents’ preferences, to the extent that they are accommodated, and when residents can rely on their collective knowledge to further direct them in the performance of ABC, trust can be developed. Such trust may convey that residents can trust in ANs’ ability to make decisions for them when they deteriorate due to the fact that the ANs are the ones who know them best [34]. According to theologian Knud Løgstrup [35], trusting in others is a natural human position, as humans trust one another until proven wrong. However, this is a serious matter, as the trusting person literally places their life in the other persons’ hands, silently asking for a loving encounter where the trust can grow. This transforms the action of showing trust into an ethical demand. Its unspoken character makes the demand radical, as it is dependent on the trusted person’s reaction. In this study, the ANs welcomed residents’ ethical demands when adaptively promoting their self-determination, be it time-consuming or causing more work to themselves, or when they sought residents’ company or offered additional ABC to residents, just in order to let them thrive. Residents appreciated this, but the building of trustful relationships with residents may also bring a sense of happiness and confidence to ANs, who may feel rewarded when achieving excellence in their work [29]. On the other hand, ANs in this study rejected the radical ethical demand when they neglected residents’ outspoken preferences, lied to them and even offended them. However, indifference is enough to reject the ethical demand raised by another person’s trust [35], i.e., when ignoring groaning and gasping during ABC. Earlier research shows that the lack of good care, while ignoring emotional aspects, may result in unmet needs and inhumane care [9]. Thus, when ANs, for various reasons, are unable to detect and respond to residents’ trust, they fail to meet their physical, psychosocial and spiritual needs, which deteriorates the quality of ABC [32]. However, what seems to be indifference may have several explanations. The loss of a dear older person who ANs have known for a long time may cause grief that conveys a risk of burnout. Thus, what looks like indifference might well be a kind of self-protection.

Nonetheless, rejecting the ethical demand is a serious error, as building trust can help residents take control by balancing their dependence on care with their desire to remain self-determined [5]. This balancing process can be meaning making in a way that makes older persons feel alive during this last period of their lives. However, a logical consequence of any ethical demand is that “ought implies can”, which means that an agent has moral obligations to perform certain actions only if it is possible for him or her to perform it [36]. Thus, if an AN suffers from mental or physical exhaustion, being incapable of showing the ethical sensitivity needed to meet residents’ ethical demand due to moral limitations, an ethical encounter is not possible. Moreover, current circumstances with low staffing and lack of time limits the possibilities for an ethical encounter. Adding to this, the AN’s joy of relatedness in caring may diminish and counteract ethical encounters [13].

From the findings of this study, some clinical implications emerge. ANs’ attitudes towards nursing home residents are complex, as they hold positive and negative attitudes concurrently. Moral deliberation and reflection are instruments and tools for understanding the close relationship between carer and patient. Therefore, opportunities for moral deliberation over the communication, compassion, decision-making and behaviour of ANs should be organised regularly in nursing homes, with consideration given to the care relationship. These initiatives may help ANs to develop greater awareness of the ethical aspects of providing ABC [37].

Nevertheless, ethical education or reflection is not enough. It is important to acknowledge the barriers preventing ANs from practising fully what their education and training have taught them. There is also a clear need for increased resources – nursing home managers, policymakers and politicians need to participate in a discussion about the apparent lack of resources and try to address and discuss these issues.

### Strengths and limitations

One methodological strength was the triangulation of multiple data sources to provide a comprehensive picture of ABC at the end of life in a nursing home. We strived to include a diverse group of participants concerning their age, gender and ethnicity, however, those who chose to participate were a rather homogenous group, which may be considered a limitation. Another limitation was that the researcher, who conducted the data collection, is a former AN and later a nurse in a nursing home context. This pre-understanding may have influenced the analysis. On the other hand, preunderstanding is an asset that helps researchers to understand the data [38]. To address this limitation, both authors analysed the data. Also, the triangulation sources may have minimised this limitation, as the stakeholders reflected critically and found the findings to be valid and trustworthy and in line with their experiences. Also, we strived to be as transparent and reflexive as possible in describing the context, data collection and analysis process.

## Conclusions

The findings in this study suggest that ethical encounters in ABC care practice in a nursing home context have many barriers that are related to communication, relationship and quality of care.The barriers included ineffective communication, a lack of resources and work values, which often hindered ethical encounters. Nevertheless, moral sensitivity, a genuine interest in resident engagement, and collaborative practices were facilitators of ethical encounters and thus central to qualified and personalised ABC. Uniquely, assistant nurses need to be aware of their responsibility and accountability for performing their tasks responsively to residents’ vulnerability. Consequently, we suggest that assistant nurses should be given opportunities for moral deliberation over issues of communication, compassion, decision-making and behavior, with particular consideration for the care relationship. Such initiatives have the prospect of helping assistant nurses to develop greater awareness of ethical aspects in ABC, provided they possess the personal prerequisites to accomplish an ethical encounter. To further improve the quality of care, such organisations must provide resources for the building of relationships, as well as time for AN’s recovery after long shifts. Moreover, there is a need for additional research, including the implementation of an ethically grounded palliative care.

## Supplementary Information

**Additional file 1.**

## Data Availability

The datasets used and analysed during the current study are available in Swedish from the corresponding author upon reasonable request.

## Abbreviations

ABC: Assisted Bodily Care
AN: Assisted Nurses
