# Supplementary figures and images for: Barriers to and facilitators of ethical encounters at the end of life in a nursing home: an ethnographic study
Source: BMC Palliat Care. 2022 Jul 23;21:134. doi: 10.1186/s12904-022-01024-0 (PMC9308208; doi:10.1186/s12904-022-01024-0)

**Fig. S1** Initial mind map from reading phase

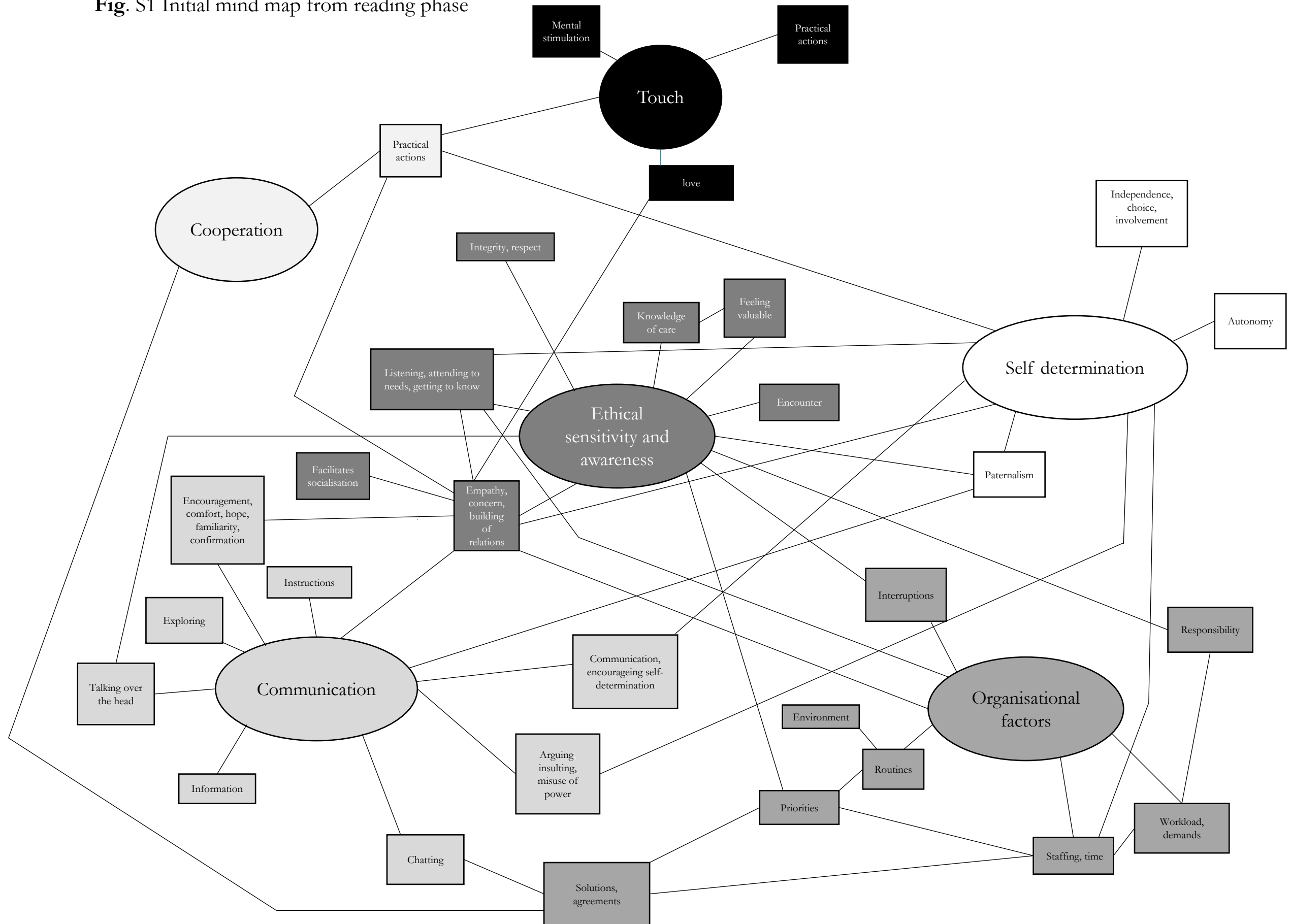

Supplement: Supplementary file 1 — Additional file 1. [file 12904_2022_1024_MOESM1_ESM.pdf]
